# Activity Diversity in Detecting Ambulatory Cognitive Deficits

**DOI:** 10.1093/geroni/igaa057.2016

**Published:** 2020-12-16

**Authors:** Jinshil Hyun, Soomi Lee, Mindy Katz, Stacey Scott, Martin Sliwinski, Richard Lipton

**Affiliations:** 1 Albert Einstein College of Medicine, Bronx, New York, United States; 2 University of South Florida, Tampa, Florida, United States; 3 Stony Brook University, Stony Brook, New York, United States; 4 Penn State University, University Park, Pennsylvania, United States

## Abstract

We examined whether participating in various daily activities (i.e., activity diversity) is associated with cognitive deficits measured by smartphone-based tests. Older adults (n=235; 70-91yrs) completed surveys 6 times/day for 14 days reporting on participation in 11 different activities (e.g., volunteering, physical activity), followed by brief ambulatory processing speed (PS) and working memory (WM) cognitive assessments. Activity diversity score was calculated using Shannon’s (1948) entropy method. Individuals’ average ambulatory PS and WM across all assessments were categorized into tertiles (i.e., high/mid/low performance). Results from multinomial logistic regression suggested that a 1SD increase in activity diversity was associated with increased odds of being in the high (better) vs. low performance tertile in PS (OR=2.1, 95%CI=[1.21, 3.51], p=.008). Activity diversity was not associated with WM. Given that cognitive deficits in PS occur earlier in the cognitive impairment process, activity diversity may be a sensitive marker for detecting very early stages of impairment.

